# Editorial: Artificial intelligence in forensic microbiology

**DOI:** 10.3389/fmicb.2023.1194390

**Published:** 2023-04-11

**Authors:** Chen Li

**Affiliations:** Research Group for Microscopic Image and Medical Image Analysis, College of Medicine and Biological Information Engineering, Northeastern University, Shenyang, China

**Keywords:** artificial intelligence, forensic microbiology, forensic science, data processing, multi-omics technique

Identifying drowning and estimating the postmortem interval (PMI) have long been challenging problems in forensic medicine. Traditional examination methods involve observing physical signs, such as white foam in the nose or mouth, lung contraction or overinflation, pulmonary edema, and the presence of water in the stomach, to determine whether death was caused by drowning. Experimental examination generally involves measuring the content of diatoms in postmortem organ tissues. Determination of the time since death still relies mainly on various corpse signs and insect development identification methods. The specificity of various signs is not strong, and diatom testing may produce false positive and false negative results. In addition, these methods are limited by the observer's experience and environmental factors, and cannot meet the requirements of accurate forensic medicine. Accurately identifying drowning as the cause of death and determining the postmortem submersion interval (PMSI) is crucial in forensic science. However, there is a pressing need to develop exact methods and indicators to accomplish these objectives with greater accuracy and specificity. In recent years, microbial research has attracted much interest among forensic professionals. Integrating next-generation sequencing (NGS) with artificial intelligence algorithms has proven to be an effective method for analyzing changes in postmortem microbial communities (Wang Z. et al.). Therefore, our aim is to explore the potential of microbiology in forensic science by focusing on the application of artificial intelligence in forensic microbiology.

In the study conducted by the research team led by Zhao (Wang L. et al.; Zhang et al., 2022), mice were divided into two groups: drowning and post-mortem submersion. Tissue samples were collected at different intervals after death, including cecal contents, liver, brain, and water, which were then amplified and sequenced using the 16s rDNA method. The research results indicated that samples taken from the brain and liver between 5 to 14 days after death are optimal for analysis. Additionally, significant differences in the microbial communities were observed in the brain and liver samples. As the PMSI increased, the dissimilarity in microbial communities between the liver and brain samples of the drowning group and the post-mortem submersion group decreased. Therefore, this method cannot be deemed reliable for determining drowning. Accurate PMSI estimation models were developed for each organ based on their microbiota. The liver had a mean absolute error (MAE) of 1.282 ± 0.189 days, the brain had a MAE of 0.989 ± 0.237d, and the cecum had a MAE of 0.818 ± 0.165d.

Similarly, in Dmitrijs' study, it was demonstrated that microbial communities can be utilized to determine the PMSI in juvenile swine (Dmitrijs et al.). In Yu's study, it was shown that artificial intelligence is better at automatically identifying diatoms in drowning cases (Yu et al.). Pan's experiment proved that there are differences in bacterial communities among different water levels in the Yellow River Basin (Pan et al.).

With the continuous advancement of detection techniques and analytical methods, we can now investigate microbial communities within samples at previously unattainable depths. Microbial communities have the potential to serve as a powerful tool for estimating the time and identifying causes of death in animal models. However, microbial communities can vary significantly depending on different environments and conditions. To promote the widespread use of microbiomes in forensic science, more research professionals must collaborate and establish a comprehensive and systematic microbial database that can be integrated with data from other omics fields.

## Author contributions

The author confirms being the sole contributor of this work and has approved it for publication.
